# Early Onset Schizo-Obsessive Disorder: A Case Series of 7 Inpatient Children

**DOI:** 10.1192/j.eurpsy.2024.929

**Published:** 2024-08-27

**Authors:** E. Yerlikaya Oral, G. Karaçetin

**Affiliations:** Department of Child and Adolescent Psychiatry, University of Health Sciences, Bakirkoy Prof. Dr. Mazhar Osman Research and Training Hospital for Psychiatry, Neurology and Neurosurgery, Istanbul, Türkiye

## Abstract

**Introduction:**

Schizo-obsessive disorder (SOD) is a complex psychiatric condition characterized by exhibiting symptoms of schizophrenia and obsessive-compulsive disorder (OCD)(Schirmbeck *et al.* Front Pharmacol. 2013 Aug 9;4:99). Some researchers prefer to describe this condition as a spectrum called “schizo-obsessive spectrum” and state that clinical represantations such as OCD with poor insight, OCD with schizotypal personality disorder, schizophrenia with obsessive-compulsive symptoms and schizophrenia with OCD are included in this spectrum(Poyurovsky *et al.* J Psychiatr Res. 2005 Jul;39(4):399-408). There is limited literature available on early on-set schizo-obsessive disorder in child and adolescent sample.

**Objectives:**

This case series aimed to describe the clinical characteristics, phenomenology, diagnostic process and treatment response of SOD in a sample of inpatient adolescents and illuminate the intricate symptomatology between schizophrenic and obsessive-compulsive features.

**Methods:**

A retrospective review was conducted of 7 adolescent patients who met DSM-V criteria for both schizophrenia and OCD in our inpatient clinic over the past year. Data were collected from medical records, including demographic information, clinical presentation, treatment history and response to treatment. All data were anonymized to maintain patient confidentiality.

**Results:**

The sample consisted of 5 females and 2 males, with a mean age of 15,4 years. All patients presented with a mixed symptomatology of hallucinations, delusions and obsessive-compulsive symptoms. Many common points observed about clinical characteristics and psychiatric history of the patients. In most of the patients, the first psychiatric complaints started with obsessive-compulsive symptoms. It was observed that obsessions evolved into over-valued ideas and delusions in the course of time. Patients responded late and inadequately to pharmacological treatment, multiple drug use was necessary. Hospitalization lasted longer, the average time was 53 days. Most of the patients required augmentation with cognitive-behavioral therapy due to partial response or intolerable side effects. Unfortunately, no patient experienced full remission or returned to premorbid functioning.

**Conclusions:**

This case series underscores the complexity of diagnosing and treating schizo-obsessive disorder in a pediatric population. It appears that a combined approach using both pharmacotherapy and psychotherapy may yield the most beneficial results. However, given the small sample size and retrospective design, these findings need to be interpreted with caution. Further research are crucial to corroborate our findings and refine treatment strategies.

**Disclosure of Interest:**

None Declared

